# Arthroscopic-assisted uniportal spinal surgery with annular repair for lumbar disc herniation in hemophilia: A case report

**DOI:** 10.1097/MD.0000000000042223

**Published:** 2025-07-18

**Authors:** Yaoyu Xiang, Jizheng Li, Xianguang Yang, Fei Sun, Xidan Hu, Tuhaopeng Shen, Jing Yang, Weiqing Ge, Tao Zhou, En Song

**Affiliations:** a Department of Sports Medicine, First Affiliated Hospital of Kunming Medical University, Kunming, Yunnan Province, China; b Department of Orthopedics, Yunnan Provincial Traditional Chinese Medicine Hospital, Kunming, Yunnan Province China; c Department of Orthopedics, Traditional Chinese Medicine Hospital of Luliang County, Qujing, Yunnan Province, China; d Clinical Pharmacy Center, First Affiliated Hospital of Kunming Medical University, Kunming, Yunnan Province, China.

**Keywords:** annular suturing repair, arthroscopic-assisted uniportal spinal surgery, hemophilia A, ligamentum flavum suspension, lumbar disc herniation, reherniation prevention

## Abstract

**Rationale:**

Lumbar disc herniation (LDH) in patients with hemophilia A (HA) presents significant surgical challenges due to elevated perioperative bleeding risks. Traditional surgical approaches may increase the likelihood of complications such as epidural hematoma and disc reherniation, necessitating innovative strategies. This report introduces arthroscopic-assisted uniportal spinal surgery (AUSS) combined with annular suturing repair and ligamentum flavum preservation as a minimally invasive approach designed to mitigate these risks and improve surgical outcomes in patients with HA.

**Patient concerns:**

A 20-year-old male presented with a 1-year history of lower back pain and 4 months of right leg pain and numbness, worsened by standing and walking. Magnetic resonance imaging and computed tomography revealed L5/S1 disc herniation compressing the right nerve root. The patient’s history of HA extended over 19 years.

**Diagnoses:**

LDH at L5/S1 and HA.

**Interventions:**

The patient underwent an AUSS with annular suture repair and ligamentum flavum suspension. Intraoperatively, the herniated nucleus pulposus was excised, and the annular defect was sutured to mitigate reherniation risk. Perioperative management included factor VIII replacement to stabilize the coagulation levels.

**Outcomes:**

Postoperatively, the patient experienced significant relief from symptoms. Follow-up magnetic resonance imaging at 1 and 6 months showed no recurrence of the disc herniation. The patient returned to normal activity without any complications.

**Lessons:**

This case illustrates that AUSS with annular suturing repair is a feasible and effective approach for treating LDH patients with hemophilia, offering minimal bleeding risk, and reduced recurrence of disc herniation.

## 1. Introduction

Lumbar disc herniation (LDH) is a common spinal disorder characterized by radicular pain, sensory deficits, and motor weakness due to nerve root compression. LDH is often linked to degenerative changes in the intervertebral disc, including nucleus pulposus dehydration, annulus fibrosus damage, and disc height reduction, which lead to annulus tears and herniation into the spinal canal.^[1,2]^ Standard LDH management involves conservative therapies, such as physical therapy, and escalates to surgical intervention when the symptoms persist.^[3]^

Conventional minimally invasive surgical options, such as microdiscectomy and percutaneous endoscopic lumbar discectomy, have shown high success in symptom relief.^[4,5]^ However, these techniques still pose unique challenges for patients with hemophilia A (HA), including a heightened risk of perioperative bleeding. HA, characterized by coagulation factor VIII deficiency, predisposes patients to significant bleeding complications during spinal surgery, increasing the risk of postoperative issues such as epidural hematoma and neurological deficits.^[6,7]^

Advanced minimally invasive techniques, such as arthroscopic-assisted uniportal spinal surgery (AUSS), have shown potential as safer alternatives for hemophilic patients by reducing surgical trauma and improving intraoperative visualization.^[8]^ Compared with traditional microdiscectomy, AUSS reduces soft tissue disruption and provides several specific advantages, including a broader operative field, improved instrument maneuverability, and convenient access to a variety of instruments. Collectively, these attributes lead to reduced intraoperative bleeding and more effective hemostasis. Moreover, AUSS enhances surgical efficiency while also serving as an optimal platform for implementing advanced surgical techniques, such as ligamentum flavum (LF) preservation and annular repair, which aim to improve the long-term outcomes. Studies indicate that preserving the LF and repairing the annulus fibrosus after discectomy can reduce the recurrence of herniation, which is particularly beneficial given the high cost of repeated factor VIII replacement in hemophilic patients.

This case report highlights the successful application of AUSS with annular suturing repair and LF preservation in a young patient with HA and LDH, demonstrating how this technique mitigates the risks associated with both spinal surgery and coagulopathy.

## 2. Case presentation

### 2.1. Patient information

A 20-year-old male patient presented with a 1-year history of progressive lower back pain and a 4-month history of radiating right leg pain accompanied by intermittent numbness. The pain was exacerbated by prolonged standing and walking, but was partially relieved with rest. The medical history was significant for HA diagnosed at the age of 1, for which the patient had not previously received regular monitoring or treatment. The patient reported no family history of HA or other bleeding disorders.

**Figure 1.**
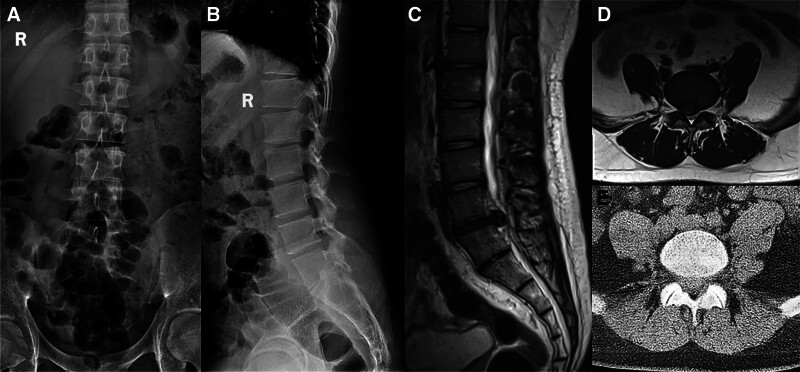
Preoperative imaging. Preoperative anteroposterior (A) and lateral (B) radiographs of the lumbar spine, showing overall spinal alignment and disc space narrowing at L5/S1. (C) Preoperative sagittal MRI T2-weighted image, demonstrating lumbar disc herniation and reduced disc height at L5/S1. Axial MRI T2-weighted image (D) and axial CT scan (E) revealing the L5/S1 disc herniation and associated nerve root compression. MRI = magnetic resonance imaging.

On physical examination, the patient exhibited a positive straight leg raise test on the right side, along with mild hypesthesia in the right L5 dermatome. Preoperative magnetic resonance imaging (MRI) and computed tomography confirmed an L5/S1 disc herniation compressing the right nerve root (Fig. 1).

The patient’s pain level was assessed as 7 out of 10 on the Visual Analog Scale, and functional impairment was rated as moderate, with an Oswestry Disability Index of 82. Diagnoses: LDH at L5/S1 and HA.

After 4 months of consistent conservative management for the LDH, which included bed rest, massage, acupuncture, and anti-inflammatory analgesic therapy, the patient experienced no significant improvement. Consequently, following multiple consultations, the patient opted for surgical intervention. Upon admission, a hematology consultation was conducted, and a perioperative coagulation management plan was established to address the bleeding risks associated with HA. This plan included daily preoperative infusions of 1000 to 1500 IU of factor VIII, beginning 3 days before surgery. The day before surgery, the dosage was increased to 2000 IU, and factor VIII levels were closely monitored at 15-minute, 30-minute, 1-hour, 4-hour, 8-hour, and 12-hour intervals postinfusion to ensure optimal coagulation.

### 2.2. Surgical technique

#### 2.2.1. Preoperative factor VIII management

To reduce the risk of hemorrhage due to hemophilia, a prophylactic dose of 2000 IU of factor VIII concentrate was administered at 8:00 am on the day of surgery. A maintenance regimen of 1000 IU was then given every 6 hours to maintain stable factor VIII levels throughout the surgery and the immediate postoperative period.

#### 2.2.2. Patient positioning and incision

**Figure 2.**
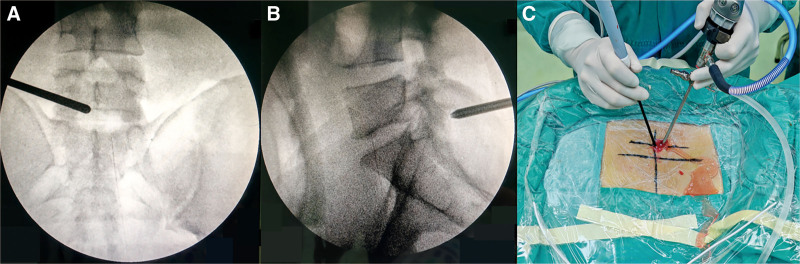
Intraoperative imaging procedures for positioning and instrumentation. (A) Anteroposterior fluoroscopic view showing the guide rod positioned at the transition between the L5 lamina and spinous process. (B) Lateral fluoroscopic view demonstrating the guide rod at the level of the L5/S1 disc space, directed toward the intervertebral foramen. (C) Placement of arthroscope and surgical instruments through a single portal to initiate exposure and decompression at the L5/S1 disc space.

The patient was placed in the prone position under general anesthesia with standard monitoring and sterile draping. Intraoperative anteroposterior fluoroscopy was used to precisely localize the L5/S1 disc level, drawing a line connecting the midpoints of the L5 and S1 pedicles on the patient’s right side parallel to the midline of the spinous processes. Lateral fluoroscopy confirmed that the entry point corresponded to the intervertebral disc space (Fig. 2A and B). Once confirmed, a midline vertical skin incision ≈1.5 to 2.0 cm in length was made along this line. The incision was considered adequately sized when it could comfortably accommodate the tip of a single index finger, allowing sufficient access while maintaining minimal invasiveness (Fig. 2C).

#### 2.2.3. Interlaminar window exposure and bone thinning

**Figure 3.**
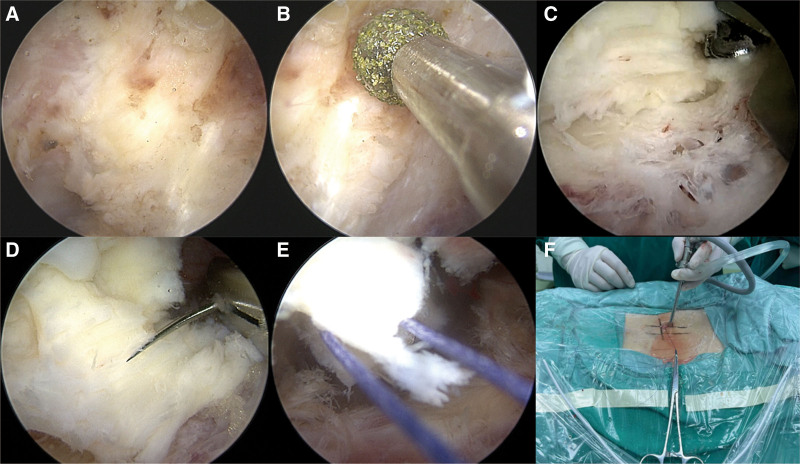
Intraoperative procedures for ligamentum flavum suspension. (A) Endoscopic view showing the interlaminar window exposure, providing clear visualization of surrounding structures. (B) Use of a high-speed burr to detach the ligamentum flavum at its insertion site, allowing access to the spinal canal. (C) Kerrison rongeur used for resection of the bony edges of the lamina, further exposing the insertion point of the ligamentum flavum to facilitate mobilization for suspension. (D) First suture placement through the ligamentum flavum for suspension. (E) The ligamentum flavum is suspended in place to improve access for decompression. (F) External view showing the ligamentum flavum held securely outside the body during the operation.

Bipolar plasma radiofrequency was used to effectively dissect the subcutaneous tissues and paraspinal muscles to ensure hemostasis. This technique minimized tissue damage and provided a clear surgical view of the interlaminar window between L5 and S1 (Fig. 3A). To facilitate the suspension and full exposure of the LF, a high-speed burr was used to carefully thin the lamina and adjacent facet joint structures, thereby providing an optimal surgical view for the following steps (Fig. 3B). This step is crucial for accessing the spinal canal and releasing the LF from its bony attachments. Bone removal provided sufficient exposure to the LF, allowing for its suspension and subsequent spinal decompression. Kerrison rongeurs were then used to resect any residual bony elements, ensuring complete mobilization of the LF for the following steps (Fig. 3C).

#### 2.2.4. LF suspension

The LF was carefully dissected and suspended to facilitate subsequent surgical steps with an emphasis on preserving its structural integrity, which is crucial for minimizing postoperative spinal instability. A 4-0 absorbable suture was passed through the lateral edge of the LF near its attachment to the facet joint (Fig. 3D). The suture was then brought externally and tensioned gently without tying, with a hemostat used to secure it, thereby maintaining adequate suspension (Fig. 3E and F).

#### 2.2.5. Herniated disc exploration and nucleus pulposus removal

**Figure 4.**
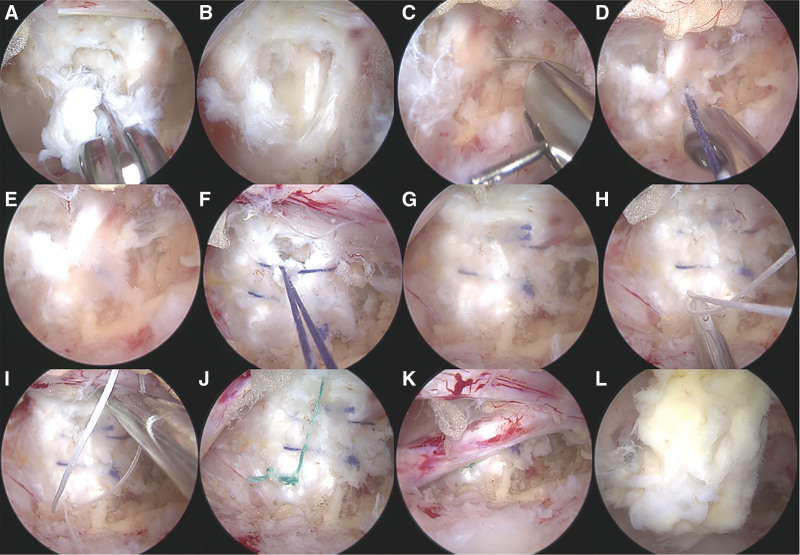
Intraoperative procedures for nucleus pulposus removal and annular repair. (A) Endoscopic view showing the removal of the extruded and loose nucleus pulposus from the L5/S1 disc space. Exposure of the annular tear (B) and placement of the first suture (C) using a 4-0 absorbable suture. Knot tying after the first suture placement (D), resulting in partial closure of the annular defect (E). (F and G) Placement of the second suture to further reduce the size of the annular tear. (H and I) Additional suturing using an annular repair device to secure the closure. Final view of the repaired annulus (J) and the overall appearance of the repair area after completion (K). (L) Repositioning of the suspended ligamentum flavum at the end of the procedure to restore its natural position.

After suspending the LF, the spinal canal was accessed for the next steps. Initially, meticulous dissection of the suspension and access to the spinal canal were established. The next step involved meticulously dissecting the surrounding epidural fat and releasing adhesions to fully expose the herniated disc. Throughout this process, bipolar plasma radiofrequency was utilized to achieve thorough hemostasis, ensuring a clear and safe surgical field. Guided by preoperative MRI findings, the spinal canal was carefully explored to identify areas of potentially causing nerve root compression. During exploration, the dura mater and nerve roots were cautiously exposed, and any compression by adjacent structures was meticulously addressed. Once the herniated disc and its relationship with the nerve structures were fully visualized, removal of the herniated nucleus pulposus was initiated. Using pituitary forceps, the protruding and loose fragments of the nucleus pulposus were gently extracted, while preserving the remaining intact disc material (Fig. 4A). This step was performed with precision to ensure that the nerve root and dura mater were fully decompressed, effectively alleviating pressure on the neural structures.

#### 2.2.6. Annular suturing repair

Following the removal of the herniated nucleus pulposus, the annular defect was clearly visualized (Fig. 4B). To restore the structural integrity of the disc and prevent future herniation, an annular suturing repair was performed using a combination of 4-0 absorbable sutures and a Smile annular suture instrument. The first suture was placed perpendicular to the annular defect, with the needle entry and exit points positioned ≈2 mm from the edges of the defect. The suture targeted the relatively healthy and structurally firm portions of the annulus fibrosus or posterior longitudinal ligament(Fig. 4C and D). Under endoscopic guidance, the suture was carefully tied and locked with a knot pusher to ensure secure closure, resulting in the partial closure of the defect with the first suture (Fig. 4E). A second suture was then placed 1 mm adjacent to the first to further reduce the size of the defect and provide additional mechanical stability (Fig. 4F and G). This double-suture technique ensures that the defect is securely closed, thereby significantly reducing the likelihood of recurrence. A Smile annular suture instrument was subsequently employed for additional reinforcement. This instrument was chosen because the remaining defect was located near the midline, where using 4-0 absorbable sutures could pose a risk of nerve root injury or puncture the dura mater. The Smile annular suture instrument offers enhanced control in this sensitive region. A white loop suture was inserted through the annulus ≈2 mm from the defect margin (Fig. 4H). A second green suture was then passed through the white loop (Fig. 4I), which was tightened to secure the knot and ensure firm closure of the annular defect. The final appearance of annular suturing repair demonstrated complete and stable defect closure (Fig. 4J and K).

#### 2.2.7. LF repositioning

Following the completion of annular suturing repair, the LF was gently released from traction and repositioned to its natural position over the dura mater and the spinal canal (Fig. 4L). Care was taken to ensure proper anatomical alignment without pressure on the neural structures, reducing the likelihood of postoperative adhesions, and preserving the ligament’s protective function. A final inspection confirmed that the surgical field was free of any residual compression or active bleeding, thus concluding this step.

#### 2.2.8. Closure

After repositioning the LF, the surgical area was inspected to ensure hemostasis. A vacuum drain was placed to prevent fluid accumulation. The skin incision, ≈1.5 to 2.0 cm, was closed in layers using absorbable sutures, followed by the application of sterile dressings. The entire procedure took ≈50 minutes, with an estimated blood loss of 20 mL. Postoperative monitoring included regular assessments to detect any immediate complications such as bleeding or neurological deficits.

## 3. Postoperative outcome

The patient demonstrated stable recovery after the procedure, supported by intensive factor VIII replacement therapy to maintain coagulation stability. During the initial 3 days, factor VIII levels were maintained at 90% to 100% activity, reducing postoperative bleeding risks, and then adjusted to sustain activity levels at 50% to 70% for the remainder of the hospital stay.

**Figure 5.**
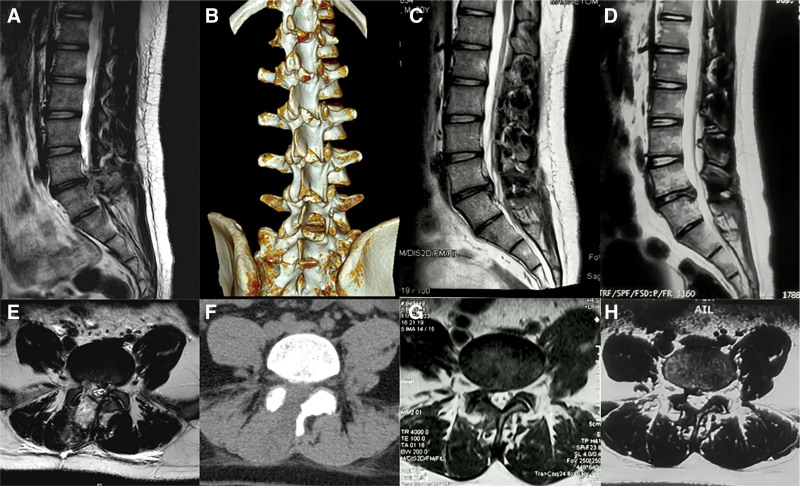
Postoperative imaging. Postoperative sagittal (A) and axial (L) MRI T2-weighted images obtained 1 week after surgery, demonstrating successful decompression at L5/S1 with preserved ligamentum flavum and proper spinal alignment. Postoperative sagittal (B) and axial (F) CT images obtained 1 week after surgery, confirming proper decompression and structural integrity at L5/S1 without any residual bone fragments or compression. Postoperative sagittal (C) and axial (G) MRI T2-weighted images obtained 1 month after surgery, showing continued maintenance of decompression at L5/S1 with no recurrence of disc herniation and clear nerve root pathways. Postoperative sagittal (D) and axial (H) MRI T2-weighted images obtained 6 months after surgery, indicating stable decompression at L5/S1, with no recurrence of herniation and clear neural structures. CT = computed tomography, MRI = magnetic resonance imaging.

In terms of pain relief and functional recovery, pain levels, assessed by the Visual Analog Scale, notably decreased from a preoperative score of 7/10 to 3/10 at 1 month postoperatively and further improved to 1/10 by the 3-month follow-up. Correspondingly, the patient’s functional status, evaluated by the Oswestry Disability Index, showed substantial enhancement from 82% preoperatively to 20% at 3 months, reflecting marked improvements in daily activities and overall quality of life. In addition, postoperative imaging confirmed the successful outcomes. One week after surgery, MRI and computed tomography (Fig. 5A, B, F, and E) showed effective decompression at L5/S1, preservation of the LF, and no residual compression. One-month MRI scans (Fig. 5C and G) showed no recurrence of the herniation. Six-month MRI (Fig. 5D and H) indicated stable decompression and no signs of reherniation or adhesions.

## 4. Discussion

This report documents the first documented AUSS combined with annular suturing repair in a patient with HA and LDH. Due to increased perioperative bleeding risks, HA poses significant challenges for spinal surgery, with potential complications, including epidural hematoma and reherniation. This case illustrates how the minimally invasive AUSS approach effectively addresses these risks, enabling a safe and successful intervention in such a complex patient profile.

The AUSS technique, also known as uniportal noncoaxial spinal endoscopic surgery, introduced by Professor En Song in 2021, offers an innovative approach to minimally invasive spinal surgery, transforming traditional open surgery into an “endoscopic open surgery” model. The AUSS maintains the standard techniques and instrumentation of open surgery while significantly reducing incision size, utilizing the arthroscope as an “extended eye” for a minimally invasive view. This approach preserves familiar operating methods, reducing the learning curve and making AUSS more accessible to spinal surgeons accustomed to open procedures. With a single, small incision (1.5–2 cm), AUSS minimizes soft tissue damage, blood loss, and recovery time. The arthroscope’s 360° rotation capability offers expansive visual access to the surgical field, reduces blind spots, and greatly enhances precision. This was particularly beneficial in this case, as it enabled thorough exploration and effective removal of herniated disc material within the spinal canal. In addition, enhanced visualization supported precise hemostasis and efficient adhesion release, which are critical for managing the complex anatomy and minimizing complications in patients with HA. AUSS’s noncoaxial endoscopic design of AUSS allows greater flexibility in tool selection and angling, accommodating a broad range of instruments, including osteotomes, Kerrison rongeurs, ultrasonic scalpels, and specialized suturing devices, thereby enhancing surgical efficiency and adaptability.^[9]^ In this case, AUSS enabled both LF suspension and annular suturing repair through a single portal, achieving stable and effective repair with minimal incision or repositioning. For patients with HA, these advantages translate to smaller incisions, precise hemostasis using multiangled radiofrequency cautery, minimal disruption of normal structures, and improved short- and long-term surgical outcomes.

Extensive fibrosis replaces the epidural fat within the spinal canal, restricting smooth gliding of the dura mater and nerve roots, which is one of the primary causes of postoperative lumbar and limb pain.^[10]^ LF provides spinal stability and elasticity while acting as a natural barrier within the spinal canal.^[11]^ In patients without significant LF hypertrophy, retaining this structure is particularly beneficial because LF preservation has been shown to reduce postoperative adhesions and improve long-term spinal health outcomes.^[12]^ Studies indicate that maintaining an intact LF reduces scar tissue formation and minimizes the risk of epidural fibrosis, which supports better functional recovery, delays degenerative changes, and preserves a clear anatomical reference for potential future surgeries.^[13,14]^ However, LF preservation has historically been challenging, with many spinal surgeons opting for removal to improve visibility during surgery. This choice is partly because of the perception that preserving the LF prolongs the operation, restricts surgical access, and complicates the procedure. In addition, in open surgeries, LF can obstruct the surgical view, making it difficult to achieve clear access to the underlying structures. In this case, the AUSS platform facilitated a novel LF suspension technique that allowed LF preservation without traditional challenges. Using the AUSS’s expansive field of view and flexible tool angling, the LF was lifted and suspended, maintaining a clear operative field without obstructing visibility or extending the operating time. This approach minimizes anatomical disruption, significantly reduces the risk of postoperative adhesions, and preserves the LF as a critical landmark for future intervention. Thus, the combination of AUSS and LF suspension offers a minimally invasive and anatomically conservative solution that aligns with modern spinal surgery principles and provides optimal surgical outcomes in high-risk patients.

The aging population has led to an increased incidence of LDH, with postoperative recurrence rates following discectomy reported as high as 3% to 25%, posing a significant challenge in achieving durable outcomes.^[15–18]^ This challenge has driven substantial research and clinical focus on techniques to enhance annular healing and thereby reduce the risk of reherniation. Despite promising advancements and research successes in gene therapy and tissue engineering for lumbar disc and annular repair, these techniques face substantial barriers to widespread clinical adoption. Barriers such as high costs, limited long-term clinical validation, issues with tissue integration and biocompatibility, specialized expertise requirements, and stringent ethical and regulatory standards collectively limit their routine clinical application.^[19–22]^ Advanced biological therapies are constrained by these practical limitations, and mechanical solutions such as surgical annular suturing have emerged as viable alternatives to prevent reherniation.^[23]^ However, surgical annular suturing, initially introduced over a decade ago, faced significant challenges that hindered its further adoption. These limitations include insufficient suture strength, which often results in further annular damage and paradoxically higher recurrence rates, as well as a high technical complexity that requires specialized skills.^[24–27]^ As a result, annular suturing has not gained traction during the development of endoscopic spinal procedures; however, recent advances in minimally invasive platforms, such as AUSS, have renewed interest in annular suturing. First, suturing the annulus has been shown to effectively reduce the risk of disc herniation recurrence, particularly within the first postoperative month – known as the peak recurrence period for nucleus pulposus herniation. Wang et al,^[28]^ in a recent study, showed that the recurrence rate within 1 year postoperatively was 0% in the annular suturing group, compared with 7.1% in the discectomy-only group. Second, many earlier limitations of annular suturing can now be mitigated by utilizing the AUSS platform. Enhanced visualization, flexibility in tool selection, and versatile operating angles provided by AUSS have significantly lowered the difficulty of performing annular suturing, reduced collateral damage, and improved suturing precision. In this case, annular suturing repair was performed using a combination of 4-0 absorbable sutures and the Smile suture device, aided by AUSS’s stable and clear operative field. This allowed precise closure of the annular defect while preserving the structural integrity of the disc, thereby preventing further extrusion of the nucleus pulposus. By maintaining the structural stability of the disc, this approach may also delay disc degeneration and spinal deterioration, thus offering long-term benefits to the patient. Annular suturing repair offers notable economic advantages, especially in patients with hemophilia. By reducing the recurrence rate and avoiding reoperations, this technique minimizes the need for costly coagulation factor VIII replacement therapy, which is essential for managing bleeding risks in this patient population. For high-risk patients, such as those with hemophilia, annular repair not only aligns with the clinical goals of reducing recurrence but also provides a cost-effective solution that reduces the financial and healthcare burdens associated with frequent reinterventions.^[29]^

The combined use of LF preservation and annular suturing repair in this case offers an innovative strategy that addresses both immediate and long-term outcomes in patients with hemophilia undergoing lumbar discectomy. For patients with HA, reducing reherniation risks is critical, as each recurrence not only necessitates reoperation but also requires costly and intensive factor VIII replacement therapy.^[30]^ By preserving the LF and ensuring annular stability, this technique aims to reduce postoperative complications, such as epidural fibrosis, mitigate mechanical stresses on adjacent discs, and potentially delay degenerative changes in the spine.^[25]^ This approach effectively aligns with the needs of hemophilia patients, enhancing surgical success and durability while reducing the financial and medical burdens of potential reoperations. Thus, the combined AUSS technique provides a sustainable, cost-effective solution that optimizes long-term spinal health and improves postoperative quality of life in high-risk patients.

While AUSS with annular repair effectively prevents immediate reherniation, it has several limitations in fully restoring the disc’s natural elasticity and load-bearing capacity, which may lead to degeneration over time – especially in younger, more active patients.^[31]^ Some patients also report only minimal postoperative improvements in pain and function, highlighting variability in long-term outcomes.^[32]^ Furthermore, the mechanical complexity of effective annular repair can limit its durability.^[24]^ In addition to these procedural limitations, this study’s single-case design inherently restricts generalizability and comprehensive efficacy validation. Despite encouraging short- to mid-term results, a longer follow-up period is necessary to assess the durability of outcomes and to detect any late-stage complications, particularly given the mechanical and biological complexities associated with annular repair in patients with hemophilia. Consequently, large-scale, long-term studies and clinical trials are essential to evaluate the sustained effectiveness of this surgical approach and validate it comprehensively. Looking ahead, integrating tissue engineering and gene therapy with AUSS-based annular suturing may enhance disc repair by regenerating biomechanical properties and restoring structural integrity. Such advances could bridge the gap between preserving spinal stability and achieving full functional recovery, potentially setting a new standard for treating LDH in high-risk patients such as those with HA.

## 5. Conclusion

The combination of AUSS with annular suturing repair demonstrated promising safety and efficacy in the treatment of LDH in a patient with HA. This approach successfully minimizes intraoperative bleeding and decreases the risk of postoperative recurrence, making it a suitable and minimally invasive option for patients with high bleeding risks. By maintaining disc stability and reducing recurrence rates, annular suturing repair not only helps reduce the dependency on coagulation factor replacement but also enhances the overall cost-effectiveness and improves long-term outcomes of the procedure.

This case offers preliminary evidence supporting the use of AUSS with annular suturing repair in high-risk populations, indicating that this strategy can significantly reduce surgical complications and recurrence rates. However, further research is warranted to explore its broader clinical implications and the potential for widespread adoption.

## Acknowledgments

The authors thank the patients for their participation in this study.

## Author contributions

**Writing – original draft:** Yaoyu Xiang.

**Writing – review & editing:** Yaoyu Xiang.

**Investigation:** Jizheng Li, Fei Sun, Xidan Hu.

**Visualization:** Xianguang Yang.

**Data curation:** Tuhaopeng Shen, Jing Yang, Weiqing Ge, Tao Zhou.

**Conceptualization:** En Song.
